# The effect of hypoglycaemia during hospital admission on health‐related outcomes for people with diabetes: a systematic review and meta‐analysis

**DOI:** 10.1111/dme.14115

**Published:** 2019-09-29

**Authors:** A. Lake, A. Arthur, C. Byrne, K. Davenport, J. M. Yamamoto, H. R. Murphy

**Affiliations:** ^1^ Cambridge University Hospitals NHS Foundation Trust Cambridge UK; ^2^ University of East Anglia Norwich Research Park Norwich UK; ^3^ Departments of Medicine and Obstetrics and Gynaecology University of Calgary Calgary Alberta Canada; ^4^ Alberta Children's Hospital Research Institute Calgary Alberta Canada

## Abstract

**Aim:**

To assess the health‐related outcomes of hypoglycaemia for people with diabetes admitted to hospital; specifically, hospital length of stay and mortality.

**Methods:**

We conducted a systematic review and meta‐analysis of studies relating to hypoglycaemia (< 4 mmol/l) for hospitalized adults (≥ 16 years) with diabetes reporting the primary outcomes of interest, hospital length of stay or mortality. Final papers for inclusion were reviewed in duplicate and the adjusted results of each were pooled, using a random effects model then undergoing further prespecified subgroup analysis.

**Results:**

In total, 15 studies were included in the meta‐analysis. The pooled mean difference in length of stay for ward‐based inpatients exposed to hypoglycaemia was 4.1 days longer [95% confidence interval (CI) 2.36 to 5.79; *I*² = 99%] compared with those without hypoglycaemia. This association remained robust across the pre‐specified subgroup analyses. The pooled relative risk (RR) of in‐hospital mortality was greater for those exposed to hypoglycaemia (RR 2.09, 95% CI 1.64 to 2.67; *I*² = 94%, *n* = 7 studies) but not in intensive care unit mortality (RR 0.75, 95% CI 0.49 to 1.16; *I*² =0%, *n* = 2 studies).

**Conclusion:**

There is an association between inpatient hypoglycaemia and longer length of stay and greater in‐hospital mortality. Studies examining this association were heterogenous in terms of both clinical populations and effect size, but the overall direction of the association was consistent. Therefore, glucose concentration should be considered a potential tool to aid the identification of inpatients at risk of poor health‐related outcomes.

## What's new?

- Heterogeneity of inpatient populations and definitions of hypoglycaemia are a significant challenge in synthesizing evidence from previously published studies.
- Hypoglycaemia events, including those considered non‐serious, are associated with an increased length of hospital stay and increased risk of inpatient mortality.
- Inpatient hypoglycaemia is a marker for clinical deterioration and potential increased risk for adverse outcomes.
- Glucose measurements could aid the identification of those at risk of poor health‐related outcomes.

**What's new?**

- Heterogeneity of inpatient populations and definitions of hypoglycaemia are a significant challenge in synthesizing evidence from previously published studies.
- Hypoglycaemia events, including those considered non‐serious, are associated with an increased length of hospital stay and increased risk of inpatient mortality.
- Inpatient hypoglycaemia is a marker for clinical deterioration and potential increased risk for adverse outcomes.
- Glucose measurements could aid the identification of those at risk of poor health‐related outcomes.

## Introduction

One in six NHS hospitals beds, across all specialties, is occupied by someone with diabetes 1. The National Inpatient Diabetes Audit reported that among 15 774 hospitalized people with diabetes in over 200 hospitals, good glycaemic control was achieved on fewer than half of their inpatient days 2. Additionally, ~ 20% of hospitalized people with insulin‐treated diabetes experience one or more episodes of hypoglycaemia, with 8% of episodes classed as severe. Hypoglycaemia is also a common occurrence in critically unwell people without diabetes.

In 2017, the International Hypoglycaemia Study Group defined hypoglycaemia in clinical trials as a glucose level of < 3.0 mmol/l, with 3.0–3.9 mmol/l redefined as an alert level. They also reported that, in people with diabetes, the counter‐regulatory response to hypoglycaemia will differ dependent on the individual's glucose control 3. It remains common practice, however, for a glucose level of < 4 mmol/l to trigger hypoglycaemia treatments in hospital 4.

Several factors are associated with the increased risk of hypoglycaemia for hospitalized people with diabetes. These may relate to changes in physiology, pharmacological treatments or the environment, and inappropriate diabetes management. Physiological and pharmacological factors include acute kidney injury, liver failure, sepsis, reduction in medications known to increase glucose levels, such as corticosteroids, and polypharmacy. Changes in the environment increase hypoglycaemia risk through factors such as the limited availability of food, long fasting time from evening meal to breakfast and unexpected deviations in hospital care. Inappropriate diabetes management includes overuse of variable rate intravenous insulin infusion, and errors in prescribing and drug administration 5, 6. Despite the potential morbidity and known risk factors for hypoglycaemia 7, measures to counteract these risks in hospital are not routinely taken.

Extant studies examining the effect of hypoglycaemia in people with diabetes who are hospitalized have generally found negative clinical outcomes, but with estimates of varying precision and from a range of clinical populations 8, 9, 10, 11, 12, 13, 14, 15, 16, 17, 18, 19, 20, 21, 22, 23, 24. Therefore, we performed a systematic review and meta‐analysis to investigate the extent to which hypoglycaemia in people who are hospitalized influences length of stay and mortality. This review focuses on people with diabetes who were exposed to hypoglycaemia during their hospital admission.

## Research design and methods

In accordance with our published protocol (PROSPERO CRD42017062611), we performed a systematic review and meta‐analysis. Studies were included if they met all the following criteria: participants were aged ≥ 16 years and had a diagnosis of diabetes (or data from study participants with diabetes could be extracted separately); the exposed group experienced hypoglycaemia (< 4.0 mmol/l or equivalent hospital coding) and was compared against a control group without hypoglycaemia; and the study outcomes included one or both of the primary outcomes for this systematic review (length of stay or mortality). Papers were excluded if study samples included participants who were admitted with hypoglycaemia, paediatric or pregnant populations, were based in a primary care or emergency department setting, or if the study did not report either outcome of interest or a qualitative research design was used. Reporting is in accordance with the Preferred Reporting Items for Systematic Reviews and Meta‐Analyses (PRISMA) guidelines 25. A multidisciplinary panel of academic and clinical experts was formed. This panel determined the review protocol and contributed to various aspects of the review.

### Data sources and search strategy

The following databases were searched for all available dates: MEDLINE (Ovid), Embase, CINAHL Complete, Scopus and Web of Science, ClinicalTrials.gov, UK Clinical trials, gateway (current and archived), Open Grey, NHS Evidence, ProQuest UK/Ireland, ProQuest International, Prospero and the Cochrane databases. These databases were chosen as they hold papers on healthcare‐related research from various professional perspectives 26, 27. The first database search was undertaken on 28 June 2017 and an updated search on MEDLINE (Ovid), Embase, CINAHL Complete, Scopus and Web of Science was undertaken on 6 June 2018 using the same search terms. Details of the search terms used are provided in Appendix S1. Both searches were undertaken through the website of the host university using the same search strategy and databases 28 ^.^

The MEDLINE (Ovid), Embase, CINAHL Complete, Scopus and Web of Science searches were carried out by the lead author and independently by an information specialist on the same day. All searches were limited to the English language. Reference management was carried out using Mendeley.

### Study selection

All titles and abstracts were assessed independently and in duplicate to identify articles requiring full‐text review against the predefined inclusion criteria. Papers found through grey literature searching were assessed by the first author (AL). Eligible citations identified after title and abstract review were all then full‐text reviewed by two people. Reasons for exclusion were recorded. Any disagreements between reviewers were resolved by consensus and in consultation with the expert panel. Review of the reference lists of the citations for full‐text review was also undertaken (by AL) to identify additional relevant papers.

### Data extraction

Data from included studies were extracted using prespecified data extraction forms. Extracted data included study demographics and design, in‐hospital location, diagnostic criteria for hypoglycaemia, sample size and outcomes reported. Where reported, adjusted findings were used. Hypoglycaemia definitions were grouped for analyses in line with the consensus recommendation made by the international hypoglycaemic group (non‐serious: ≥ 3.0 and < 4.0 mmol/l; serious: < 3.0 mmol/l) 3. Authors were contacted if studies had missing data or inconsistencies. If the data could not be retrieved or queries resolved, the citation was excluded from the meta‐analysis. The standard deviation was not reported for two studies and was therefore imputed based on the mean of the studies reporting a similar mean. These publications were removed during sensitivity analysis 29. Record management was carried out using Microsoft Excel^®^.

### Data synthesis

The primary outcome was hospital length of stay and all‐cause mortality. Data were pooled into relative risk (RRs) or mean difference with 95% confidence intervals (CI) for dichotomous and continuous outcomes, respectively. Meta‐analysis was performed using random effects models, applying the DerSimonian and Laird statistical method 30.

A prespecified analysis was undertaken stratified by hospital location (ICU, medical wards, speciality areas or not specified), research methodology, hypoglycaemia definition, removal of outliers, removal of poor‐quality papers, prospective cohort study design and time point of reported outcomes (in hospital mortality vs. post discharge mortality). Statistical heterogeneity was assessed through the *I* ^2^ test for heterogeneity. Regression tests for analysis and publication bias were not completed because there were fewer than 10 papers overall for publication bias or per covariate in meta‐regression tests 31, 32. Small study effect was examined using funnel plots. Analysis was conducted using RevMan version 5.3.

### Quality assessment

Studies were reviewed to determine whether the cohort observed was representative of the study population and whether there was risk of bias in the recruitment process or identification of the exposure to hypoglycaemia 33. Bias and methodological assessment was completed by two reviewers (AL and CB) using an adapted version of the Newcastle–Ottawa scale 34. The Newcastle–Ottawa scale rates eight items relevant to cohort study design: representativeness of cohort, selection of non‐exposed cohort, ascertainment of exposure, demonstration of no exposure at admission, comparability of cohorts, assessment of outcome, sufficient length of follow‐up and adequacy of follow‐up. Each study was then given a total score equating to a rating of poor, fair or good quality. The results were compared, and disagreements resolved through consensus 35.

## Results

**Figure 1:**
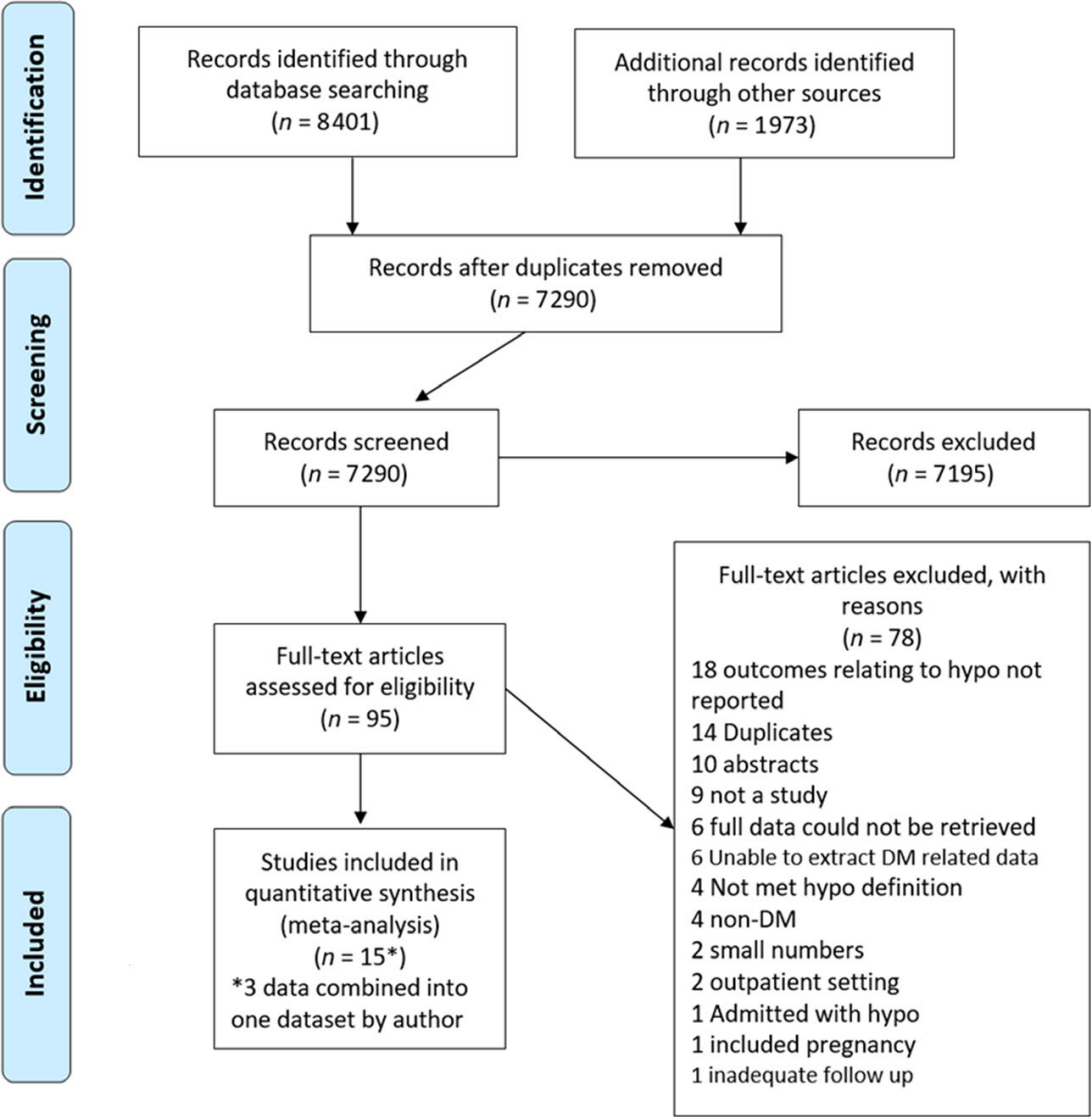
PRISMA flow chart.

A total of 10 374 papers were identified, 8401 through database searches and 1973 from other sources. Once duplicates had been removed, 7290 papers remained and were reviewed by title and abstract for eligibility. Some 7195 studies were excluded based on the inclusion criteria, leaving 95 for full review. A further 72 studies were excluded following full‐text review, leaving 23 for inclusion. Another six were excluded as the data required for analysis could not be extracted. For length of stay, the reason for exclusion was typically because a mean and standard deviation could not be extracted. For mortality, the most common reason for exclusion was because data for people with diabetes could not be separated from data for those without. Of the 17 remaining studies, three papers were by the same author Krinsley 8, 9, 10 and a combined data set was kindly provided by the lead author to prevent duplication of analysis. This resulted in 15 eligible studies 8, 9, 10, 11, 12, 13, 14, 15, 16, 17, 18, 19, 20, 21, 22, 23, 24 (Fig. 1).

### Study characteristics

The characteristics of the 15 included studies are shown in Table 1. The hospital locations included a general ward (*n* = 6), ICU (*n* = 4), location not specified (*n* = 4) and a specialty cardiac ward (*n* = 1). Studies were published between 2009 and 2016 and the designs included observational studies (*n* = 9), sub‐analysis of a randomized controlled trial (RCT) (*n* = 3), nested cohort within a RCT (*n* = 1), sub‐analysis of a cross‐sectional study (*n* = 1) and a case‐controlled study (*n* = 1). Geographical locations included the USA (*n* = 7), UK (*n* = 2), and one study each from Spain, the Netherlands, Saudi Arabia, Singapore, Italy and Sweden. Most were single‐centre studies with a mean sample size of 32 788 (range 288 to 300 020), with a mean of 11 425 people with diabetes exposed to hypoglycaemia. All studies adjusted for age, sex and comorbidities. Eight studies adjusted for at least one other patient demographic such as socio‐economic status, ethnicity, health insurance status or education level.

**Table 1:** Eligible studies included in the meta‐analysis

Lead author, Year	Country	Hospital location	Study design	Total patients or admissions	Total exposed to hypoglycaemia	People with diabetes (%)	Tytpe 1 diabetes	Type 2 diabetes	Treated with insulin (%)	Treated with oral agents (%)	Non‐exposed group	Adjustments during analysis	Hypoglycaemia definition (mmol/l)	Outcome
Gomez‐Huelgas, 2015 11	Spain	N/S	RCS	309 020	154 510	100	N/S^d^	291 827	N/S	N/S	MP	Age, sex, type of diabetes, readmission, CCI, primary or secondary hypoglycaemia	ICD code	↑LOS, ↑ In‐hospital mortality
McEwan, 2015 12	UK	N/S	RCS	2158	1079	100	424	1734	100	0	MP	Age, sex, type of diabetes, use of medication, BMI, HbA_1c_, smoking, geographical region and CCI	ICD code	↑LOS, ↑In‐hospital mortality^a^
Curkendall, 2009 13	USA	N/S	RCS	103 813	8234	100	5261^d^	87 610^d^	25.5	N/S	NM	Age, sex, type of diabetes, race, specific complications, events and conditions during hospitalization^d^, CCI, insulin use, hospital characteristics and clinically plausible interactions	< 3.9	↑LOS, ↑In‐hospital mortality
Geremakis, 2014 14	USA	N/S	CCS	8952	2510	100	N/S	N/S	N/S	N/S	CC	Ethnicity, admitting hospital, CCI, surgery, fungal infection, antipsychotics, pulmonary hypertension, antidepressants, beta adrenergic, cephalosporin, antibiotics	<2.2	↑LOS
The NICE‐SUGAR Study Investigators, 2012 15	USA	ICU	SaRCT	6026	643	20	N/S	N/S	N/S	N/S	NM	Age, sex, APACHE II score, BMI, blood glucose, postoperative status, sepsis, trauma, diabetes, prior insulin or steroid treatment, cardiac failure, intensive vs. conventional insulin	≤ 3.9	↑Mortality within 90 days
Krinsley, 2007, 2011, 2017 8, 9, 10	USA	ICU	SaRCT	2786^b^	683	100	N/S	N/S	N/S	N/S	NM	Age, sex, APACHE II score, APACHE III score, mechanical ventilation	< 3.9	↑In‐hospital mortality
Sechterberger, 2013 16	Netherlands	ICU	RCS	10,320.	57	16	N/S	N/S	98	N/S	NM	Age, sex, APACHE II score, hypoglycaemia severity, cardiothoracic surgery, glucose variability, mean glucose, glucose levels ≤ 4.7 mmol/l	≤ 2.2	↑ICU mortality
Arabi, 2009 17	Saudi Arabia	ICU	NC	523	46	40	N/S	N/S	87	N/S	NM	Age, sex, BMI, postoperative status, APACHE II score, diabetes, admission glucose, mechanically ventilated, vasopressor, sepsis, creatinine, dialysis/filtration, intensive insulin	≤ 2.2	→ ICU mortality
Turchin, 2009 18	USA	General ward	RCS	4368 admissions	338 admissions	100	N/S	N/S	61.8% received insulin, OHA or both		NM for mortality. MP for LOS	Age, sex, ethnicity, health insurance, weighted mean daily glucose, LOS based on DRG and modified CCI	≤ 2.8	↑LOS ↑In‐hospital mortality
Ong, 2015 19	Singapore	General ward	RCS	288	54	100	N/S	N/S	91% received insulin, OHA or both		NM	Age, sex, ethnicity, BMI, HbA1c, number of comorbidities, systolic BP, admitting specialty, diabetes treatment, steroids and IV dextrose	<4.0	↑LOS
Borzi, 2016 20	Italy	General ward	SaCSS	3167	385	100	0	3167	N/S	N/S	NM	Age, sex, BMI, concomitant disease, insulin treatment, serum creatinine, HbA_1c_, fasting glucose and number of treatments other than diabetes	< 3.9	↑LOS ↑In‐hospital mortality
Nirantharakuma 2012 21	UK	General ward	RCS	6374 admissions	648 admissions	100	N/S	N/S	25	N/S	NM	Age, sex, ethnicity, deprivation, admission type, insulin use, modified CCI	< 3.9	↑In‐hospital mortality, ↑ LOS^c^
Kim, 2014 22	USA	General ward	RCS	1276 admissions	313	99	63^d^	1198^d^	0	100	NM	Age, sex, ethnicity, insulin use, CCI	≤ 3.9	↑LOS
Boucai, 2011 23	USA	General ward	RCS	31 970^b^	1717	34	N/S	N/S	19	11	NM	Age, sex, ethnicity, co‐morbidities, number glucose determinators, diabetes treatment	≤ 3.9	↑In‐hospital mortality, ↑LOS
Mellbin, 2008 24	Sweden	Cardiac speciality	SaRCT	1253	153	100	0	1253	N/S	N/S	NM	Age, sex, smoking status, previous myocardial infarction and coronary interventions, cardiac failure, pharmacological treatment, creatinine, diabetes duration, blood glucose before and during admission	< 3.0	→Total mortality (median follow up 2.1 years)

a Applicable only to people with diabetes (not included in meta‐analysis as data not available).

b Data provided by author.

c Not in meta‐analyses as only median data reported.

d Remainder had unknown or undisclosed type of diabetes.

N/S, not specified; ICU, intensive care unit; RCS, retrospective cohort study; CCS, case controlled study; SaRCT, sub‐analysis of randomised control trial; NC, nested cohort within a RCT; SaCSS, sub‐analysis of two cross‐sectional studies; OHA, oral hypoglycaemic agents; MP, matched patients; NM, non‐matched; CC, case controlled; CCI, Charlson comorbidity score; DRG, diagnostic related group; LOS, length of stay.

### Quality assessment

Of the 15 studies reviewed, the ascertainment of hypoglycaemia exposure and selection of the non‐exposed cohort were clearly reported. Many studies that would otherwise have scored highly were rated poor because it was not explicitly stated whether the population was not admitted with hypoglycaemia or the exact follow‐up duration. We recognize that this is likely due to many of the included studies not being published with their suitability for subsequent systematic review in mind. All papers reported the selection of the non‐exposed cohort, method of ascertainment of exposure and used record linkage for assessment of outcome.

### Association between inpatient hypoglycaemia and length of hospital stay outside intensive care

**Figure 2:**
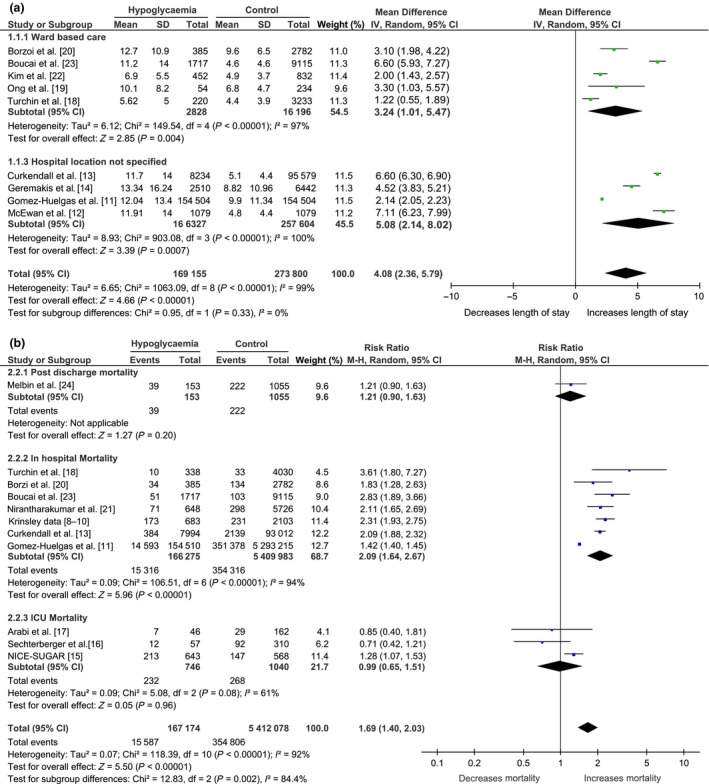
Forest plots of (a) mortality and (b) length of stay.

The overall pooled mean difference of the nine studies reporting length of stay outcomes 11, 12, 13, 14, 18, 19, 20, 22, 23 suggests that people with diabetes, who experienced at least one episode of hypoglycaemia during their admission, had an increased length of hospital stay by a mean of 4.08 days (95% CI 2.36 to 5.79; *n* = 9 studies) (Fig. 2a). This statistically significant association held for all subgroup analyses. Although the statistical heterogeneity was very high (*I*² = 99%), all included studies demonstrated that hypoglycaemia was associated with increased length of stay even when only the five papers reporting non‐serious definitions of hypoglycaemia were included (4.37 days, 95% CI 2.13 to 6.61; *I*² = 98%, *n *= 5 studies) 13, 19, 20, 22, 23.

### Association between inpatient hypoglycaemia and all‐cause mortality by mortality time point

The overall pooled relative risk (RR) of in‐hospital mortality from the ward‐based or location not specified studies 11, 13, 18, 20, 21, 23, 24 was 1.90 (95% CI 1.51 to 2.39; *I*² = 93%, *n* = 7 studies) suggesting that the risk of in‐hospital mortality is nearly doubled for people with diabetes exposed to hypoglycaemia. This remained significant when analysis was restricted to the two studies reporting 90‐day and post‐discharge mortality (RR 1.26, 95% CI 1.08 to 1.47; *I*² = 0%, *n* = 2 studies) 15, 24. However, there was no association found for the overall pooled RR of the two studies reporting ICU mortality (RR 0.75, 95% CI 0.49 to 1.16; *I*² = 0%, *n* = 2 studies) 16, 17.

The in‐hospital mortality results were obtained in the presence of substantial statistical heterogeneity (Fig. 2b). This heterogeneity was reduced, and the risk further increased when only in‐hospital mortality and non‐serious hypoglycaemia definitions were included (RR 2.15, 95% CI 1.98 to 2.33; *I*² = 0%, *n* = 5 studies) 8, 9, 10, 13, 20, 21, 23 (Table 2). Of the papers looking at the association with a serious hypoglycaemia episode (< 3 mmol/l), only one reported in‐hospital mortality 18. Throughout the subgroup analyses, the association between hypoglycaemia and mortality remained statistically significant outside ICU mortality.

**Table 2:** Summary of subgroup analysis for the effect of inpatient hypoglycaemia on length of stay and mortality

Category	Studies included (*N*)	Result^a^	*I*² (%)
Subgroup analysis of the effect of hypoglycaemia on length of stay			
All studies	9	4.08 (2.36 to 5.79)	99
Removal of papers rated as poor quality	4	3.59 (0.80 to 7.62)	99
Removal of non‐cohort studies	7	4.15 (2.11 to 6.19)	99
Removal of studies with imputed standard deviation	6	3.62 (2.09 to 5.14)	98
General ward location only	6	3.24 (1.01 to 5.47)	97
Hospital location not specified only	4	5.08 (2.14 to 8.02)	100
Inclusion of non‐serious hypoglycaemia definitions only	5	4.37 (2.13 to 6.61)	98
Inclusion of serious hypoglycaemia definitions only	2	2.87 (‐0.36 to 6.10)	98
General ward areas only and removal of outliers	8	2.14 (1.30 to 2.99)	70
General ward location and removal of papers rated as poor quality	2	1.63 (0.87 to 2.40)	67
Hospital location not specified, and removal of papers rated as poor quality	2	5.58 (3.55 to 7.62)	97
Subgroup analysis of the effect of hypoglycaemia on mortality			
All studies	11	1.69 (1.40 to 2.03)	92
Removal of papers rated as poor quality	5	1.81 (1.36 to 2.42)	90
Removal of non‐cohort studies	4	1.62 (1.16 to 2.26)	95
Removal of studies with imputed data	9	1.57 (1.29 to 1.91)	87
In‐hospital mortality only	7	2.09 (1.64 to 2.67)	94
In‐hospital mortality and non‐serious hypoglycaemia definition	5	2.15 (1.98 to 2.33)	0
Intensive care unit mortality only (all used serious hypo definitions)	2	0.75 (0.49 to 1.16)	0
90‐day and post discharge mortality only	2	1.26 (1.08 to 1.47)	0

a Mean difference in length of stay in days or risk ratio (95% confidence interval).

### Small study effect

The studies reporting length of stay did not take the inverse funnel shape expected suggesting the potential presence of publication bias (Fig. S1). Although publication bias is less problematic in the mortality analyses, there is some potential for publication bias within the in‐hospital mortality subgroup (Fig. S2).

## Discussion

This is the first meta‐analysis pooling the reported data on the effect of inpatient hypoglycaemia exposure on length of hospital stay and all‐cause mortality. We found an overall positive association between hypoglycaemia and both increased length of stay and in‐hospital mortality outside ICU settings. No significant association was found between ICU mortality and hypoglycaemia. This nonsignificant finding could reflect the frequency of blood glucose monitoring, and more responsive treatment of hypoglycaemia in ICU compared with the general ward setting. Additionally, the population treated within an ICU setting are likely to be fundamentally different from those cared for on a hospital ward. None of the included studies within the ICU setting reported length of stay.

The strengths of this review include a comprehensive search strategy, designed and tested with an information specialist, reviewers’ specialist diabetes knowledge and inclusion of experienced systematic review researchers. Limitations include the poor‐quality rating for some studies, the presence of substantial clinical and statistical heterogeneity, and the inability to be sure that people were not admitted primarily for diabetes‐related acute events other than hypoglycaemia (diabetic ketoacidosis and hyperglycaemic hyperosmolar state). We were also unable to eliminate language bias or control for different methods of glucose measurements (capillary vs. venous), glycaemic variability, causes of hypoglycaemia and in‐hospital hypoglycaemia management.

Despite these limitations, there are good reasons to be confident in these findings. They are supported by publications that were considered for inclusion but not eligible for the meta‐analysis 36, 37, 38, 39, 40. Furthermore, our findings support the statements from the American Diabetes Association 41 and the Joint British Diabetes Societies 4 guidelines who report that hypoglycaemia increases risks among people with diabetes admitted to hospital.

This review has demonstrated some of the complexities and challenges associated with diabetes inpatient research. The main areas of methodological and clinical heterogeneity were the varying definitions for hypoglycaemia, different hospital locations and different research methodologies used. We attempted to address these as robustly as possible through pre‐specified sensitivity testing. During acute inpatient hospital care, people from a variety of backgrounds are brought together in a hospital environment, with various presenting conditions ranging from routine surgery (e.g. cataracts, varicose vein) to life‐threatening emergencies (e.g. aortic aneurysm, peritonitis). Controlling for heterogeneity among this diverse population is not possible. Even within hospital location and diabetes type, individual health needs and disease severity vary greatly. This is likely to be a contributing factor to the substantial statistical heterogeneity. However, because the positive association between hypoglycaemia exposure and increased length of stay was largely consistent, we believe that the statistical heterogeneity represents variance in magnitude rather than the direction of the overall association.

Papers included within this review were published before the new biochemical definition of hypoglycaemia in clinical trials was published in 2017 3. As result, many of the studies included in their definitions of hypoglycaemia events that would now be considered an ‘alert level’. We attempted to address this through subgroup analysis. Contrary to expectations, the association between non‐serious hypoglycaemia and length of hospital stay and in‐hospital mortality remained. However, the definition of non‐serious hypoglycaemia used in some studies may have included those exposed to serious hypoglycaemia as this was not an exclusion 8, 9, 10, 13, 15, 19, 20, 21, 22, 23.

Whether hypoglycaemia has a causal effect or is a marker of ill health is unclear and outside the scope of this review. One theory for the finding that hypoglycaemia at alert levels (≥ 3.0 and ≤ 4.0 mmol/l) within the hospital setting is associated with increased risk, is that the early associated counterregulatory response to hypoglycaemia could be harmful in acutely unwell people with diabetes. Catecholamine release in response to hypoglycaemia is less well tolerated by older patients and those with cardiac morbidities. In addition, hypoglycaemia increases both platelet aggregation and prothrombotic factors, which could also contribute to increased harm 42, 43. Although the association may not be causal, this review suggests it could be a marker for ill health or worse outcomes.

Hypoglycaemia and fear of potential hypoglycaemia remain a major barrier for healthcare professionals when supporting people with diabetes in hospital to achieve optimal glucose control 44, 45, 46. Inadequate education and clinical knowledge about the risks associated with and strategies for prevention of hypoglycaemia in hospitalized people, may contribute to the suboptimal inpatient diabetes management documented by successive National Inpatient Diabetes Audits 1. This review supports the need to be cautious in balancing the risks of hypoglycaemia with optimal hyperglycaemia management. To achieve better inpatient diabetes control, the gap between evidenced‐based medicine and clinical practice needs to be considered carefully. Consideration could be given to including hypoglycaemia on early warning systems, such as the national early warning score to raise awareness and prompt a timely review of glucose management, or document glucose alongside other vital signs used to detect clinical deterioration 47, 48.

More research is required to gain a deeper understanding of the barriers to, and potential strategies for, providing optimal inpatient diabetes care. More work is needed to update non‐specialist healthcare professionals to implement best care for people with diabetes while admitted to hospital and support increasingly time pressured front‐line hospital staff to have timely access to evidence.

## Funding sources

A. L. conducts independent research supported by the National Institute for Health Research (MRES‐2015‐03‐025‐204). The views expressed in this publication are those of the authors and not necessarily those of the NHS, the National Institute for Health Research or the UK Department of Health.

## Competing interests

None disclosed

## Supporting information


**Appendix S1**. Final search strategy used in MEDLINE.
**Figure S1**. Hypoglycaemia and length of stay funnel plot.
**Figure S2**. Hypoglycaemia and mortality funnel plot.
