# Supplementary material for: The effect of hypoglycaemia during hospital admission on health‐related outcomes for people with diabetes: a systematic review and meta‐analysis
Source: Diabet Med. 2019 Sep 29;36(11):1349–59. doi: 10.1111/dme.14115 (PMC7004204; doi:10.1111/dme.14115)

**The effect of hypoglycaemia during hospital admission on health-related outcomes for people with diabetes: a systematic review and meta-analysis**

A. Lake, A. Arthur, C. Byrne, K. Davenport, J. M. Yamamoto and H. R. Murphy

Appendix S1 - Final search strategy used in MEDLINE

Ovid MEDLINE (R) 1946 to June Week 3 2017

1. Diabetes Mellitus.mp. or exp Diabetes Mellitus/

2. diab*.mp.

3. 1 or 2

4. exp Hospitalization/

5. hospitali*.mp.

6. 4 or 5

7. Hypoglycemia/ or hypoglycemia.mp.

8. Hypoglycaemia.mp.

9. (low adj2 glucose).mp.

10. (low adj2 sugar).mp.

11. 7 or 8 or 9 or 10

12. 3 and 6 and 11

13. Limit 12 to English language

Figure S1. Hypoglycaemia and length of stay funnel plot


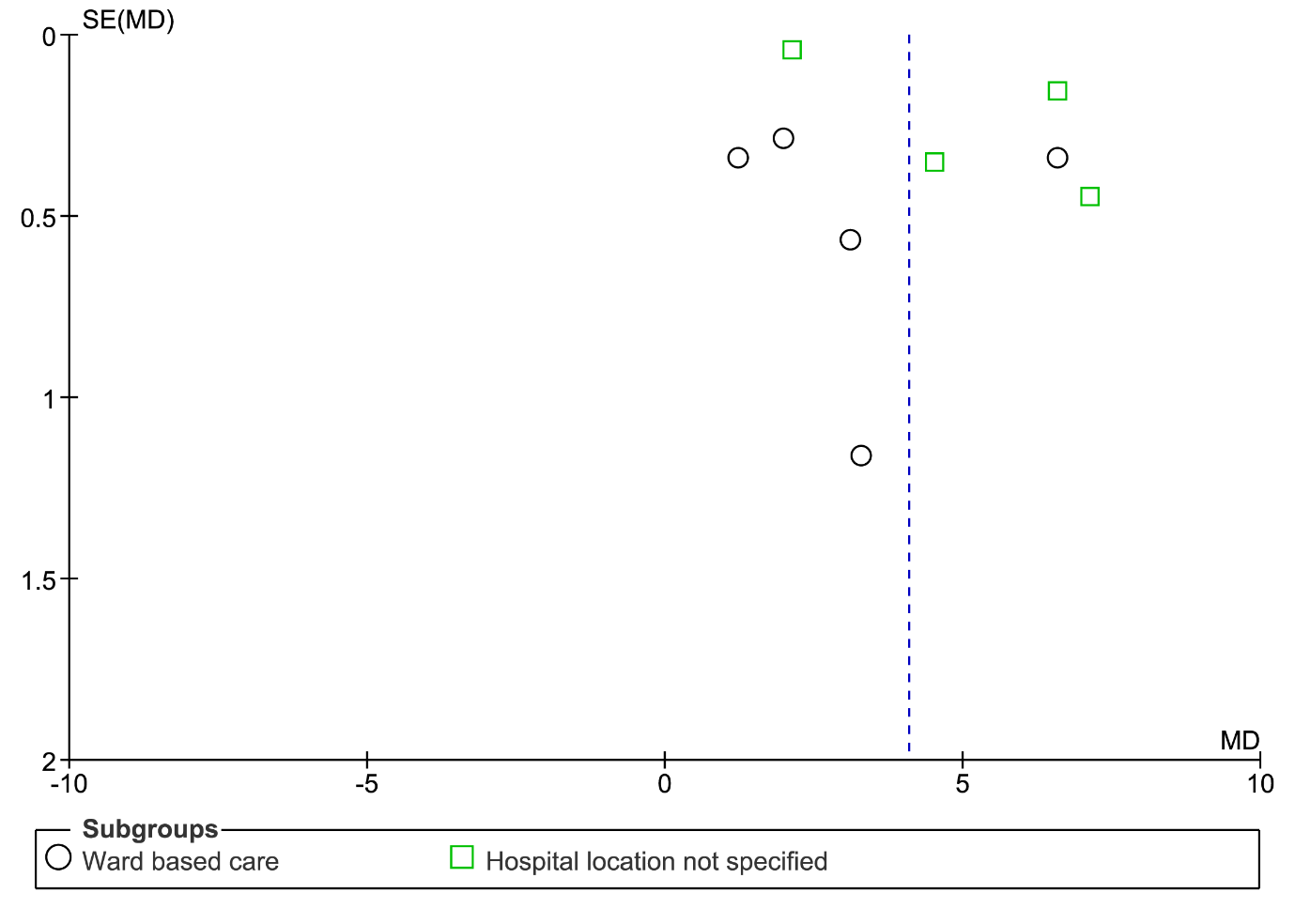


Figure S2 Hypoglycaemia and mortality funnel plot


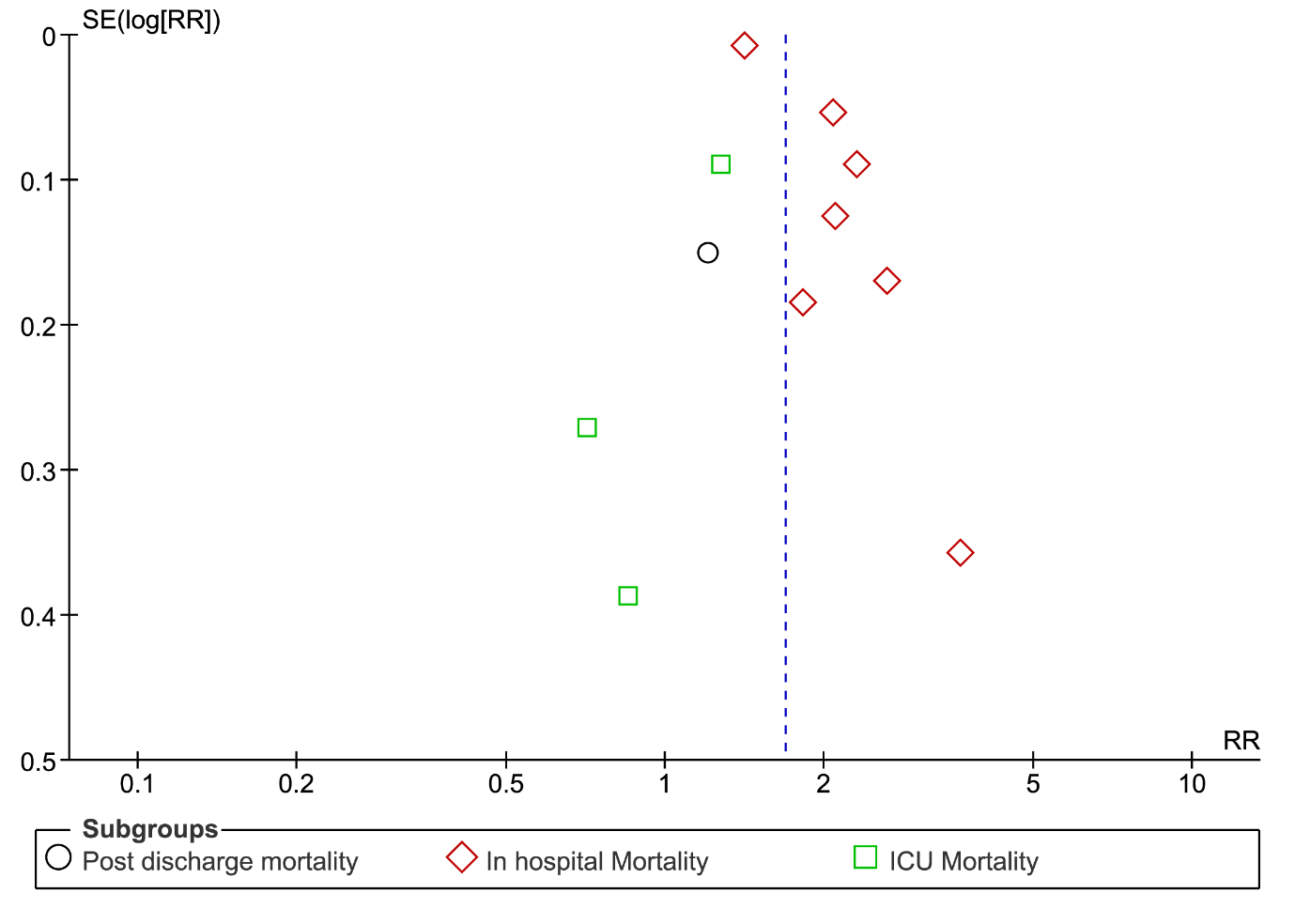

Supplement: Supplementary file 1 — Appendix S1. Final search strategy used in MEDLINE. Figure S1. Hypoglycaemia and length of stay funnel plot. Figure S2. Hypoglycaemia and mortality funnel plot. [file DME-36-1349-s001.docx]
